# Net Positive Charge of HIV-1 CRF01_AE V3 Sequence Regulates Viral Sensitivity to Humoral Immunity

**DOI:** 10.1371/journal.pone.0003206

**Published:** 2008-09-12

**Authors:** Satoshi Naganawa, Masaru Yokoyama, Teiichiro Shiino, Takeyuki Suzuki, Yoshiaki Ishigatsubo, Atsuhisa Ueda, Akira Shirai, Mitsuhiro Takeno, Satoshi Hayakawa, Shigehiro Sato, Osamu Tochikubo, Shingo Kiyoura, Kaori Sawada, Takashi Ikegami, Tadahito Kanda, Katsuhiko Kitamura, Hironori Sato

**Affiliations:** 1 Department of Public Health, Yokohama City University School of Medicine, Kanagawa, Japan; 2 Center for Pathogen Genomics, National Institute of Infectious Diseases, Tokyo, Japan; 3 AIDS Research Center, National Institute of Infectious Diseases, Tokyo, Japan; 4 Department of Internal Medicine and Clinical Immunology, Yokohama City University Graduate School of Medicine, Kanagawa, Japan; 5 College of Nursing, Yokohama City University School of Medicine, Kanagawa, Japan; 6 Department of Pathology and Microbiology, Nihon University School of Medicine, Tokyo, Japan; 7 Department of Bacteriology, Iwate Medical University, Iwate, Japan; 8 SGI Japan, Ltd, Tokyo, Japan; 9 Ryoka Systems Inc., Tokyo, Japan; AIDS Research Center, Chinese Academy of Medical Sciences and Peking Union Medical College, China

## Abstract

The third variable region (V3) of the human immunodeficiency virus type 1 (HIV-1) envelope gp120 subunit participates in determination of viral infection coreceptor tropism and host humoral immune responses. Positive charge of the V3 plays a key role in determining viral coreceptor tropism. Here, we examined by bioinformatics, experimental, and protein modelling approaches whether the net positive charge of V3 sequence regulates viral sensitivity to humoral immunity. We chose HIV-1 CRF01_AE strain as a model virus to address the question. Diversity analyses using CRF01_AE V3 sequences from 37 countries during 1984 and 2005 (*n* = 1361) revealed that reduction in the V3's net positive charge makes V3 less variable due to limited positive selection. Consistently, neutralization assay using CRF01_AE V3 recombinant viruses (*n* = 30) showed that the reduction in the V3's net positive charge rendered HIV-1 less sensitive to neutralization by the blood anti-V3 antibodies. The especially neutralization resistant V3 sequences were the particular subset of the CCR5-tropic V3 sequences with net positive charges of +2 to +4. Molecular dynamics simulation of the gp120 monomers showed that the V3's net positive charge regulates the V3 configuration. This and reported gp120 structural data predict a less-exposed V3 with a reduced net positive charge in the native gp120 trimer context. Taken together, these data suggest a key role of the V3's net positive charge in the immunological escape and coreceptor tropism evolution of HIV-1 CRF01_AE *in vivo*. The findings have molecular implications for the adaptive evolution and vaccine design of HIV-1.

## Introduction

The third variable region (V3) of human immunodeficiency virus type 1 (HIV-1) envelope gp120 subunit participates in determination of viral infection coreceptor tropism [1], [2]. It is usually composed of 35 amino acids, which form a loop-like structure on the gp120 monomer [3], [4]. The V3 and the conserved outer domain of gp120 create the binding surface for viral infection coreceptors after the binding of gp120 to the primary infection receptor CD4 [4], [5]. These interactions and successive conformational changes of gp120 are essential in rendering the initially occluded hydrophobic domain of the envelope gp41 subunit available to fusion with cellular plasma membrane.

The HIV-1 V3 is highly variable. In parallel with the V3 sequence variation, many types of infection coreceptors are reported. These are the members of the G protein-coupled receptor superfamily. The two most common types of infection coreceptors in humans are the CC chemokine receptor 5 (CCR5) and the CXC chemokine receptor 4 (CXCR4) [6]. Notably, a single group of the HIV-1 variants using the CCR5 (R5 virus [6]) predominates during the first several to 10 years or more of persistent infection *in vivo* [7], [8]. Other tropism variants including CXCR4-tropic variants (X4 virus [6]) can grow at early stage of infection by needle stick injuries, but are replaced with the R5 viruses after seroconversion [9], [10]. They generally grow better only during progression to AIDS. The R5 and X4 viruses are distinguishable by sequence feature of V3: the R5 V3 amino acid sequences generally have a lower net positive charge than those of X4 [3], [11]. Only a few basic substitutions in V3 can switch the viral coreceptor tropism from CCR5 to CXCR4 [12], [13]. Considering the extremely high levels of mutation rate of HIV-1, these findings suggest that strong selective forces are continually purifying the R5 viruses during long-lasting persistent infection.

The HIV-1 V3 is highly immunogenic, tolerant to change, and variable presumably to evade immune recognition [14]–[16]. HIV-infected individuals make high levels of anti-V3 antibodies that are reactive with soluble, monomeric gp120 protein [17], [18]. However, they often react poorly or only with low affinity to the native, oligomeric form of the gp120 protein [17], [18]. The inaccessibility of the oligomeric envelope protein is particularly prominent in the primary HIV-1 isolates [19]–[21], which are usually the R5 viruses. Indeed, studies with limited set of viruses have shown that antibodies reactive with the R5-virus V3s tend to bind to the monomeric but not the oligomeric gp120s [22], [23], and they poorly neutralize the R5 viruses [23]–[25]. In contrast, antibodies against X4-virus V3s usually bind to both forms of gp120s [22], [23], and they potently neutralize the X4 viruses [23]–[25]. Consistent with the lower sensitivity of R5 viruses to anti-V3 antibody neutralization, positive selection for amino acid variation is less prominent in the R5 virus V3 sequences, and V3 amino acid sequences of the R5 virus are relatively homogeneous among virus isolates [26], [27] or in infected individuals [28]–[30] compared with those of the X4 viruses.

While the immunological escape, variation, and coreceptor tropism evolution of HIV-1 is an important issue from both clinical and scientific viewpoints, current studies are largely confined to those of HIV-1 subtype B from North America and Europe. In this study, we attempted to obtain and integrate information on HIV-1 CRF01_AE strain [31] circulating in Southeast Asia. Specifically, we examined whether net positive charge of HIV-1 CRF01_AE V3 sequence regulates viral sensitivity to humoral immunity. Here, we demonstrate by combining bioinformatics, experimental, and protein modelling approaches that the reduction in net positive charge of HIV-1 CRF01_AE V3 sequence reduces viral sensitivity to humoral immunity and simultaneously confers viral CCR5 tropism. The findings suggest a key role of the V3 net positive charge in the immunological escape, variation, and the coreceptor-tropism evolution of HIV-1 CRF01_AE *in vivo*.

## Results

### Correlation of HIV-1 CRF01_AE V3 net positive charge, V3 prevalence, and V3 diversity

A previous case study has suggested that a group of CRF01_AE V3 sequences for the viral CCR5 tropism is resistant to the selective force for amino acid variation [29], [30]. To extend this finding with three infected individuals, we conducted large-scale analysis of V3 diversity using public database information. V3 sequences of the CRF01_AE strain were extracted from the HIV Sequence Database (http://www.hiv.lanl.gov/content/hiv-db/mainpage.html). A single V3 amino acid sequence per infected individual was randomly extracted. Sequences with ambiguous bases are excluded from the analysis. V3 sequences (*n* = 1361, 35 amino acid length) from 37 countries during 1984 and 2005 (see supporting information Figs. S1a and S1b) were used for the diversity analysis. The 1361 V3 sequences were divided into two subsets, “a” and “b”, which lack and have the glycosylation motif, respectively. Each group was divided into subgroups on the basis of the net charge; arginine, lysine, and histidine were counted as +1, aspartic acid and glutamic acid as −1, and other amino acids as 0.

**Figure 1:**
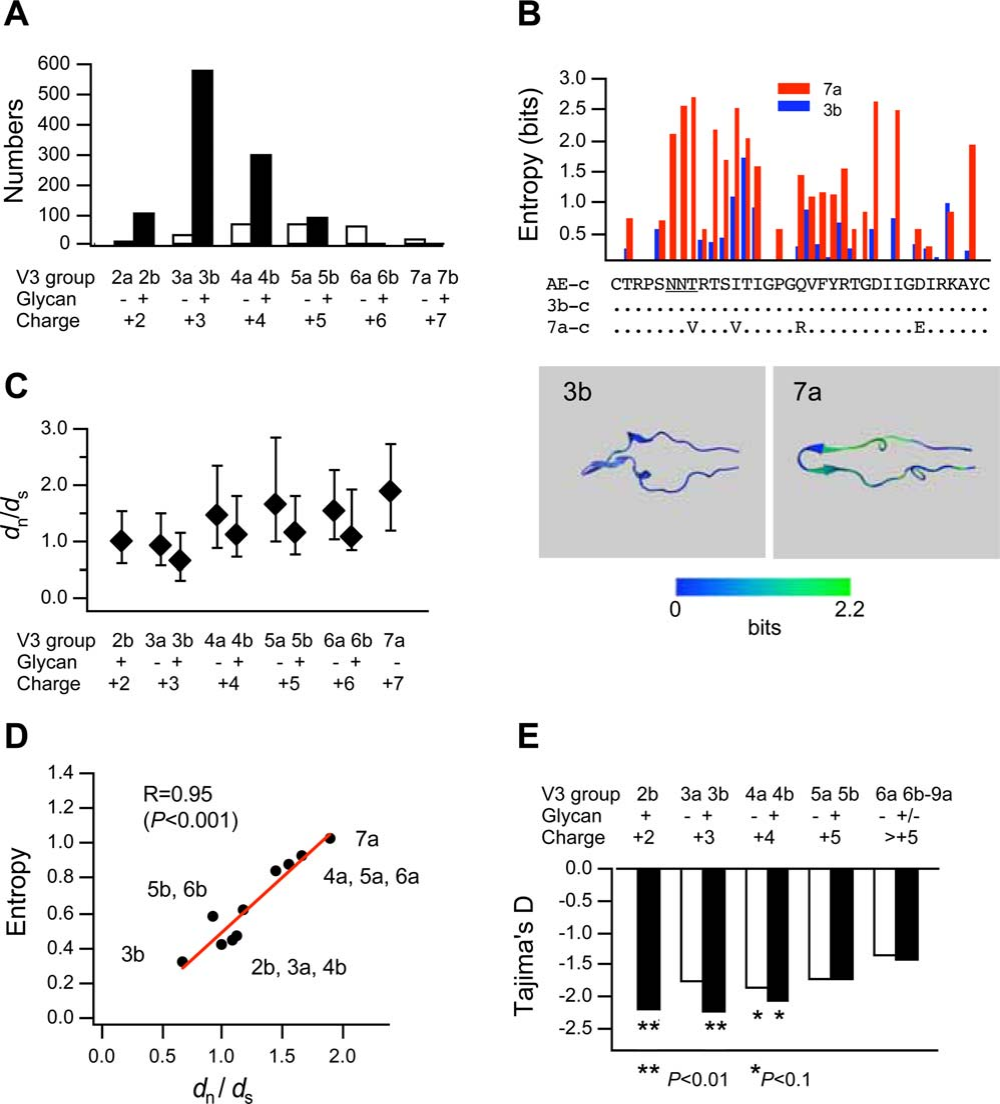
V3 net positive charge influences V3 diversity. HIV-1 CRF01_AE V3 sequences (*n* = 1361) were grouped on the basis of net positive charge and glycosylation capability *(A)* Distribution of the V3 structural variants of HIV-1 CRF01_AE in the public database. *(B)* Shannon entropy scores [3] on primary and three-dimensional structures of 3b and 7a V3s. AE-c, 3b-c, and 7a-c indicate consensus sequences for all CRF01_AE sequences, 3b V3 group (*n* = 576), and 7a V3 group (*n* = 21), respectively. *(C)* Median (diamond) and interquartile range (vertical bar) of ratios of *d*_n_/*d*_s_, *(D)* Relation of median *d*_n_/*d*_s_ ratios and average Shannon entropy scores, and *(E)* Tajima's D statistic values [32] for each V3 structural group.

Although there are exceptions, the V3 amino acid sequences capable of directing the viral CCR5 tropism of the CRF01_AE strain generally have net positive charges of +2 to +4 and the conserved N-linked glycosylation motif (asparagine-X-threonine/serine) at positions 6 to 8 [29], [30]. Consistent with the dominance of the R5 viruses in humans, less positively charged, glycosylated V3 sequences for the CCR5 tropism (2b, 3b, and 4b) were dominant in the database for over 15 years, independent of the sampling period (Fig. 1A and Fig. S1c). Shannon entropy scores representing amino acid variation were relatively low for the most abundant 3b V3 compared to those for the 7a V3 for the CXCR4 tropism (Fig. 1B), consistent with previous report [3]. Nucleotide substitutions for amino acid change were more suppressed in the V3s for the CCR5 tropism compared with those for the CXCR4 tropism (Fig. 1C). The 3b V3 had the lowest ratio of nonsynonymous to synonymous substitutions (*d*_n_/*d*_s_) with about 0.6, and acquisition of a glycosylation site decreased the *d*_n_/*d*_s_ ratios (*P* = 0.001, Table S1). The *d*_n_/*d*_s_ ratios correlated positively with the Shannon entropies, with lower *d*_n_/*d*_s_ ratios for lower entropies (Fig. 1D). Similar effects of the net positive charge of V3 on V3 diversity were detected in other major genetic lineages of HIV-1 circulating in the world, such as subtypes A, B, and C (Fig. S2).

If the low levels of amino acid changes in the V3 structures for CCR5 tropism involved the elimination of new mutants in natural selection, negative values for Tajima's D statistic would be expected [32]. Indeed, Tajima's D statistic was significantly negative for 2b and 3b V3s (*P* = 0.01, Fig. 1E and Table S2). Together, these findings on V3 diversity provide further evidence that the V3 sequences for the CCR5-tropism are less variable in nature due to the limited positive selection for amino acid diversity compared with those for CXCR4 tropism.

### Correlation of HIV-1 CRF01_AE V3 net positive charge, HIV-1 neutralization sensitivity, and HIV-1 coreceptor tropism

A positive selection pressure for the V3 diversity can be the humoral immunity. To examine whether the V3 net positive charge regulates HIV-1 neutralization sensitivities to the anti-V3 antibodies, we used V3 recombinant viruses (*n* = 30). The recombinant viruses have the CRF01_AE V3s in the backbone of the X4 virus gp120 of HIV-1 subtype B, LAI strain [13], [30], [33]. The V3s were from HIV-1 proviral DNA clones in the peripheral blood mononuclear cells of three infected individuals at the asymptomatic stage or AIDS [13], [30], [33]. These V3s could be grouped into the 2b (*n* = 2), 3b (*n* = 4), 4b (*n* = 5), 5b (*n* = 3), 6b (*n* = 5), 3a (*n* = 1), 4a (*n* = 2), 5a (*n* = 3), 6a (*n* = 3), and 7a (*n* = 2) sequences (Fig. S3). The 2b, 3b, and 4b V3 clones were the most prevalent in the three infected individuals examined, and their sequences were mostly identical [29], consistent with the V3 prevalence and diversity data in this study. The neutralization sensitivity of each recombinant virus was assessed with a single-round viral infectivity assay [34]. In parallel, titers of plasma antibody reactive with the V3 elements of the recombinant viruses were measured with V3-peptide-based, enzyme-linked immunosorbent assay (ELISA) [35].

**Figure 2:**
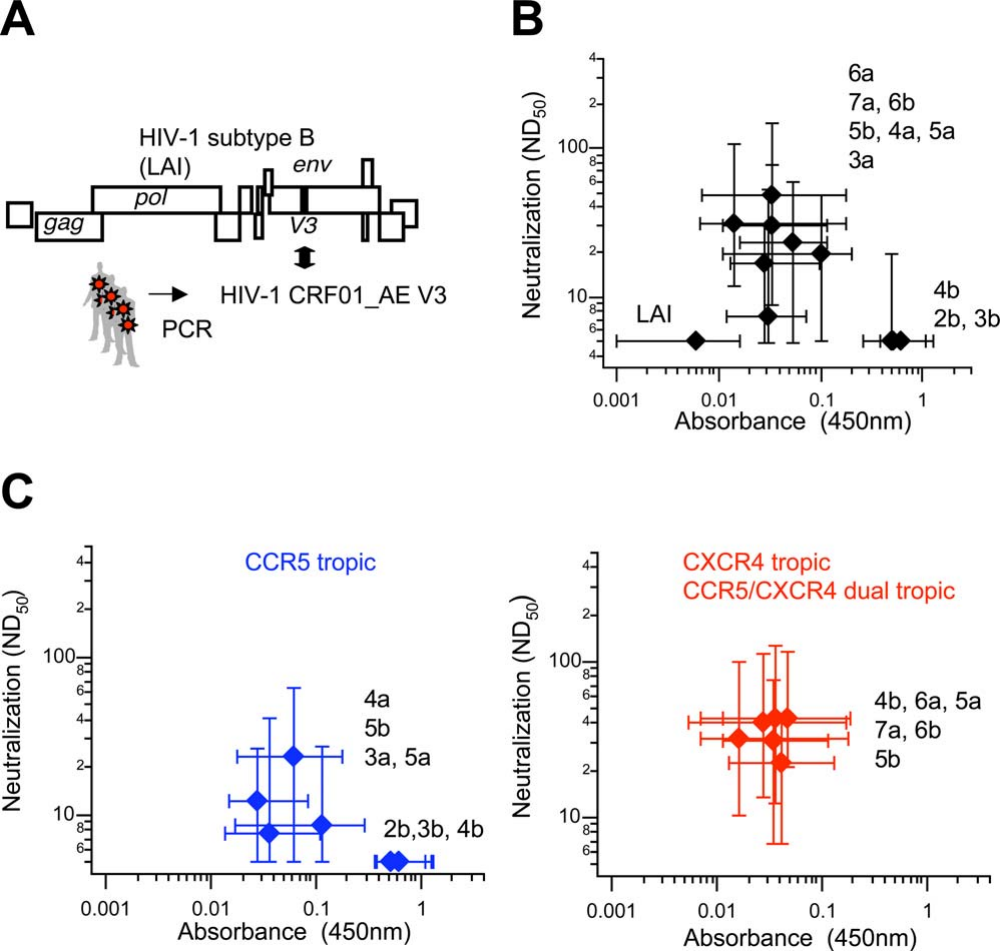
Reduction in V3 net positive charge causes loss of HIV-1 neutralization sensitivity to blood antibodies against V3 PND. *(A)* Genome structure of the V3 recombinant viruses (*n* = 30) [13], [30], [33]. *(B)* Blood anti-V3 antibody titers and viral neutralization sensitivity to the blood antibodies. Plasma samples (*n* = 20) were obtained from CRF01_AE positive individuals. Plasma antibody binding activities to the synthetic peptides corresponding to each V3 PND of the recombinant viruses were measured by V3-peptide-based ELISA [35] (Absorbance at 450 nm). The same plasma samples were used to measure ND_50_ against recombinant viruses in a single-round infectivity assay using CD4^+^CXCR4^+^CCR5^+^ HeLa cells [34] (Neutralization). Medians (diamond) and interquartile ranges for individual V3 structural groups are shown. *(C)* Neutralization sensitivity and coreceptor tropism. The recombinant viruses were grouped into CCR5-tropic (left) and CXCR4-tropic (right) variants using data reported previously [13], [30], [33].

When the V3 synthetic peptide of the parental virus of the recombinant viruses was used for the immunoassay, the CRF01_AE plasma samples had only traces of binding antibodies (Fig. 2B, Absorbance of LAI). The blood samples failed to neutralize this virus (Fig. 2B, ND_50_ of LAI). Thus, the blood samples tested are lacking in anti-V3 binding antibodies, as well as neutralization antibodies against the parental subtype B virus. The results agree with low levels of V3 amino acid identities between subtype B and CRF01_AE strains (http://www.hiv.lanl.gov/content/hiv-db/mainpage.html). They also agree with the assumption that V3 sequence diversity causes neutralization escape of HIV-1.

All blood samples from CRF01_AE infected individuals contained antibodies that bound to the synthetic peptides from the CRF01_AE strain V3 sequences of the recombinant viruses (Fig. 2B, Absorbance of 2b to 7a). Coincidentally, they neutralized a group of the viruses having particular V3 sequences (Fig. 2B). The neutralization-sensitive viruses had V3s lacking a glycosylation site (3a, 4a, 5a, 6a, 7a, *n* = 11), or V3s having a glycosylation site and increased net positive charge (5b and 6b, *n* = 8) (Fig. S3). The neutralization activities were abrogated by protein G pre-treatment of the plasma samples (Fig. S4), showing that neutralization is indeed mediated by antibodies in the plasma samples.

Notably, the blood samples poorly neutralized a group of V3 recombinant viruses (Fig. 2B, ND_50_ of 2b, 3b, and 4b, *n* = 11). These viruses had weakly charged V3s and had an N-glycosylation site, which are the characteristics of V3s for CCR5 tropism (Fig. S3, V3 IDs of 2b, 3b, and 4b). The lack of neutralization activities was not due to the lack of anti-V3 binding antibodies against these recombinant viruses. The blood samples contained high levels of antibodies that bound to the 2b, 3b, and 4b V3 peptides, more so than of other V3 groups (Fig. S5 and Fig. 2B, Absorbance of 2b, 3b, and 4b). The results are consistent with the high levels of prevalence and limited diversity of these V3 sequences (Fig. 1). The study shows that the group of V3 elements for CCR5 tropism is highly immunogenic, whereas binding antibodies raised in humans generally show only weak neutralization activities. This neutralization-resistant phenotype associated with particular V3 group was observed reproducibly in a multiple-round infectivity assay, suggesting that the phenotype is intrinsic to the viruses (Fig. S6).

The relation of this neutralization-resistant phenotype and HIV-1 coreceptor tropism was examined using information on coreceptor usages of the V3 recombinant viruses [13], [30], [33]. Importantly, the V3s having the viral-resistant phenotype unexceptionally rendered HIV-1 CCR5-tropic (Fig. 2C, left panel, V3 groups of 2b, 3b, and 4b, *n* = 10). On the other hand, V3s conferring CCR5 tropism did not always render HIV-1 resistant (Fig. 2C, V3 groups of 3a, 4a, 5a, and 5b, *n* = 7). Thus, the V3s associated with the neutralization resistance are a subset of the V3 elements associated with viral CCR5 tropism. In contrast, all V3s associated with viral CXCR4 tropism and CCR5/CXCR4 dual tropism rendered HIV-1 susceptible to neutralization (Fig. 2C, V3 groups of 4b, 5a, 5b, 6a, 6b, and 7a, *n* = 13).

The group of viruses having 4b V3s were neutralization-resistant only when they had the CCR5-restricted tropism (Fig. 2C, 4b in left and right panels). The 4b V3s associated with viral neutralization resistance had no basic substitutions compared with the CRF01_AE consensus (Fig. S3, recombinant IDs of A1, A2, A4, and A5). By contrast, the neutralization-sensitive version had two basic substitutions (Fig. S3, recombinant ID of B10). The results may imply that V3 basic substitutions at particular positions in addition to the overall net positive charge play a critical role in the determination of viral neutralization sensitivity and coreceptor tropism.

We further examined whether a CCR5-tropic but not a CXCR4-tropic envelope of CRF01_AE strain is linked to viral resistant to neutralization by anti-V3 antibodies. For this purpose, we used a pair of nearly isogenic R5 and X4 virus clones that have 3b and 5a V3, respectively [36], [37]. The results obtained with these clones and 35 blood specimens were consistent with present study and indicated that the CCR5-tropic but not the CXCR4-tropic envelope protein of the HIV-1 CRF01_AE strain was linked to viral resistance to neutralization with anti-V3 antibodies in the blood (data not shown).

### HIV-1 V3 net positive charge and V3 conformation

To obtain molecular insights into the roles of the V3 net positive charge in regulating HIV-1 neutralization sensitivity, we conducted computer-aided structural analysis. Currently, X-ray structure information on the HIV-1 R5 virus gp120 monomer bound with soluble CD4 [3] is available in the Protein Data Bank. With the data, we attempted to obtain a gp120 monomer structure for the pre-CD4 binding stage to address initial V3 conformation before receptor interaction. We first constructed gp120 outer domain models of the V3 recombinant viruses used in this study by a homology modelling method. Molecular dynamics (MD) simulation was then performed with the homology models.

**Figure 3:**
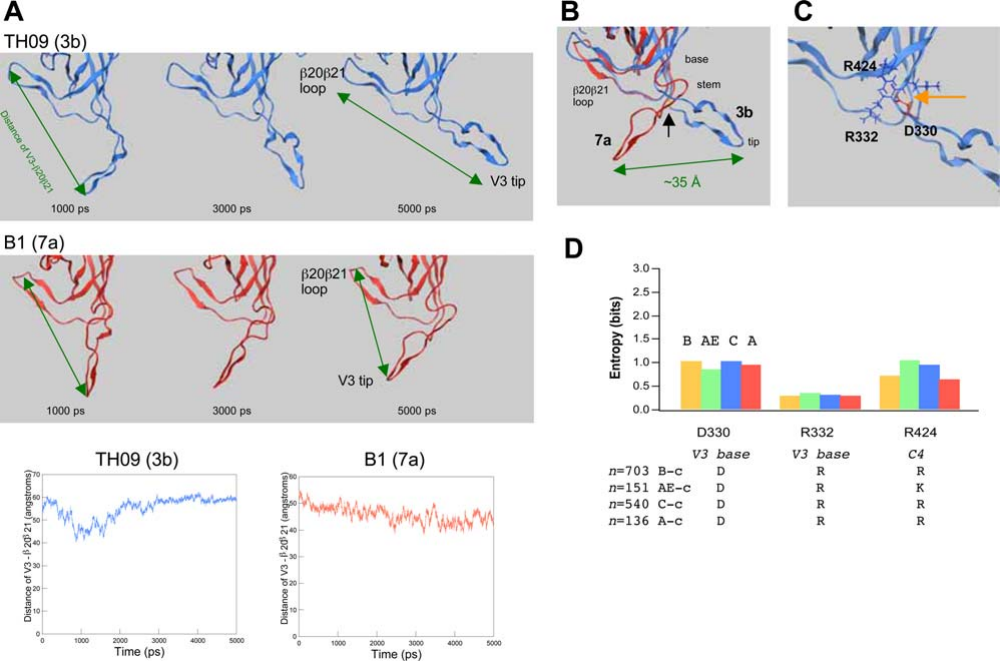
MD simulation of the HIV-1 gp120 outer domain. The V3 subset conferring the neutralization-resistant phenotype is referred to in this study as rV3: it has net positive charges of +2 to +4, an N-glycosylation site, and a capability to direct viral CCR5 tropism. The non-rV3 renders HIV-1 more susceptible to blood antibody neutralization. It has net positive charges of greater than +4 and a capability to direct viral CXCR4 tropism. *(A)* Examples of the MD simulation of two recombinant virus p120 outer domains with rV3 (TH09) and non-rV3 (B1). Distance between the Cα atom of P318 at the V3 tip and the Cα atom of Q433 at the β20β21 loop were monitored for 5 nanoseconds. *(B)* Superimposition of the gp120 monomers with the TH09 V3 (blue) or B1 V3 (red) at the simulation time of 5 nanoseconds. *(C)* Close-up view of the base-stem region of the TH09 V3. Orange dotted lines around the tip of the orange arrow indicate three hydrogen bonds at the V3 base. *(D)* Shannon entropy scores of the amino acids at the positions of 330, 332, and 424 in the public database. The positions in the gp120 of the HIV-1_LAI_ [48] are used for the amino acid numbering.

Figure 3A shows examples of the MD simulation of two recombinant virus gp120s with 3b and 7a V3 elements. The TH09 V3 (3b V3) is from an HIV-1 CRF01_AE infected asymptomatic patient, identical to the CRF01_AE V3 consensus sequence (Fig. S3, recombinant ID of TH09), rendered HIV-1 neutralization-resistant and CCR5-tropic (Fig. 2C, left panel). The B1 V3 (7a V3) is from an AIDS patient, more positively charged (Fig. S3, recombinant ID of B1) and rendered HIV-1 neutralization-sensitive and CXCR4-tropic (Fig. 2C, right panel).

The MD simulations show that V3 configuration is nearly equilibrated up to 5 nanoseconds of simulation times (Fig. 3A). Notably, the TH09 V3 was equilibrated at a much more distant position from the β20β21 loop in the outer domain than the B1 V3 (Figs. 3A and B). Hydrogen bonds were formed around the TH09 V3 base between D330 and R332, and D330 and R424, which contributed to stabilizing the V3 configuration (Fig. 3C). However, the hydrogen bonds were not formed with the gp120 having the B1 V3 (Fig. S7). Coulombic repulsion between B1 V3 and R424 increased about 44-fold as compared with that of TH09 V3, with electrostatic energies of +2.0 and +0.045 kcal/mole for B1 and TH09, respectively. The repulsion was greatest on the R424 residue in the gp120 outer domain. The results suggest that an increase in the V3 net positive charge influences electrostatic balance at the V3 base.

Importantly, the amino acids around the V3 base are relatively conserved in nature. The D330 and R332 are located at the V3 base and highly conserved within each subtype of the HIV-1 M group in the public database (Fig. 3D). The conservation was seen even in the V3s for the CXCR4 tropism (Fig. 1B). The R424 is in the fourth constant region (C4) of the gp120 core, and neighbouring amino acids are also conserved. The CRF01_AE strain alone has lysine at position 424, whereas K424 is conserved within the CRF01_AE. These data suggest that most HIV-1 gp120 monomers have the potential to stabilize V3 configuration at the base and that basic amino acid substitutions in V3 have strong influences on the V3 configuration.

**Figure 4:**
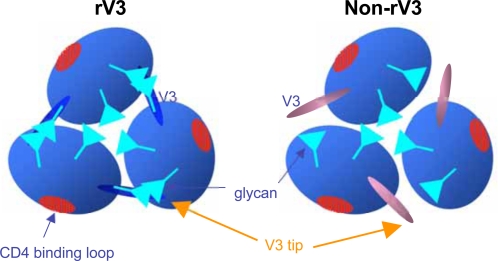
Models for the self-directed masking of V3 by mutations for the CCR5 tropism. The MD data in Fig. 3 and the HIV-1 gp120 trimer structure from cryoelectron microscopy [38] were used to construct the gp120 trimer models with CCR5-tropic (left) or CXCR4-tropic (right) V3. The models were made so that the MD data and experimental data [3], [4], [38] are compatible.

The MD simulation data were incorporated into those of a gp120 trimer structure obtained by cryoelectron microscopy [38] to illustrate schematically the V3 position in the native gp120 trimer (Fig. 4). Some glycans are also schematically illustrated at the appropriate regions. The models predict that less positively charged V3 protrudes into the outer domain of the neighboring gp120 monomer, whereas V3 with increased net positive charge protrudes away from the neighboring monomer in the trimer context.

## Discussion

HIV-1 is the causative agent of AIDS and is responsible for more than 2 million deaths every year. Understanding the immunological escape, variation, and coreceptor tropism evolution of HIV-1 is critical for developing strategies for anti-HIV interventions. In this regard, current studies are largely confined to those of HIV-1 subtype B from North America and Europe. In this study, we focused on the study of HIV-1 CRF01_AE strain circulating in Southeast Asia. The HIV-1 CRF01_AE is one of the five major HIV-1 subtypes circulating in the world [31] and thus an important strain for public health of Asia, as well as of world. However, much less basic information are available as compared with the HIV-1 subtype B.

We first demonstrate with bioinformatics approach using 1361 sequences in public database that CRF01_AE V3's net positive charge influences V3 diversity and prevalence. We found that the net positive charge of V3 influences V3 diversity (Fig. 1). Acquisition of the N-glycosylation motif in V3 augmented the sequence conservation. Our data of *d*_n_/*d*_s_ ratios and Tajima's D statistic provide strong evidence that the reduction in V3 diversity is due to limited positive selection for amino acid changes. These findings are compatible with previous findings with subtype B that sequence diversity is smaller among R5 virus V3s [26]–[28]. Moreover, the findings demonstrate the generality of findings obtained with an intra-familial infection case of CRF01_AE infection [29], [30]. The evidence that the V3 net positive charge influences V3 diversity is not solely based on sequence analysis. Structural and neutralization data suggest that V3 net positive charge regulates V3 diversity by controlling V3 structure and neutralization sensitivities, as discussed below.

We next examined potential causes of the differential diversity of CRF01_AE V3 sequences. We demonstrated with neutralization assay of V3 recombinant viruses that the V3 net positive charge influences HIV-1 neutralization sensitivity. We found that reduction in the net positive charge of V3 caused reduction in viral neutralization sensitivity to the blood anti-V3 antibodies in infected humans (Fig. 2). Again, acquisition of the N-glycosylation motif in V3 augmented the effect. We further confirmed that the especially neutralization resistant V3 sequences all render HIV-1 CCR5 tropic (Fig. 2C). These results are compatible with previous findings with subtype B that R5 viruses are more refractory to anti-V3 antibodies [23]–[25]. Together with sequence analysis data, these findings suggest that anti-V3 antibodies can act as a positive selection pressure to increase V3 sequence diversity and that V3 positive net charge can influence V3 diversity by regulating neutralization sensitivity to the V3 antibodies.

Importantly, present data revealed that not all the CCR5-tropic V3 sequences render HIV-1 resistant to the neutralization. Some V3 sequences lacking N-glycosylation site or those have the glycosylation site but have net positive charge of +5 conferred CCR5 tropism on HIV-1, whereas they were relatively sensitive to the antibody neutralization (Fig. 2C). These data are consistent with findings on CRF01_AE V3 sequence diversity and prevalence in this study. Together, the data suggest that a particular subset of CRF01_AE V3 for CCR5 tropism confers a selective advantage on HIV-1 in the face of humoral immunity and that the anti-V3 antibodies may be an important selective force to maintain CCR5-tropic V3 sequences with limited amino acid changes during persistent infection.

We further examined molecular mechanisms of neutralization escape. We found with MD simulation that reductions in the net positive charge of V3 caused a shift of V3 position in the gp120 monomer (Fig. 3). This is the first indication that the net positive charge of V3 regulates V3 configuration in the gp120 monomer. By incorporating our MD data into experimental data of HIV-1 gp120 structures [3], [4], [38], we proposed a model of V3 masking that explains how the net positive charge of V3 regulates HIV-1 V3 neutralization sensitivity (Fig. 4). The model explains present diversity and neutralization data: it predicts that less positively charged V3 for CCR5 tropism positions in the gp120 trimer context such that it protrudes into the outer domain of the neighboring gp120 monomer, which will inevitably result in better protection of V3 from antibodies by the glycans. This model also explains why anti-V3 antibodies bind effectively to monomeric but not native, oligomeric form of the gp120 protein of the HIV-1 R5 viruses [22]–[25]. Further structural studies are under way to assess the model.

Our study provides molecular insights into the mechanisms of coreceptor-tropism evolution of HIV-1. Due to high levels of viral mutation rate, vigorous and continual viral replication *in vivo*, and viral tolerance to V3 mutations, many viable HIV-1 V3 mutants would be continuously generated during a persistent infection. Therefore, why only the R5 virus dominates during persistent infection is a long-lasting question in HIV-1 research. Our study suggests a selective advantage of a subset of CCR5-topic V3 sequences in the face of humoral immunity: self-masking of neutralization epitope by reduction in net positive charge. Higher levels of the *d*_n_/*d*_s_ ratios for more positively charged V3 sequences for CXCR4 tropism (Figs. 1C and D) may imply that the X4 viruses might persist as a minority, continually receive positive selection pressures for amino acid changes, and outgrow only when the host immunity is severely damaged.

It will be important to examine why neutralization resistant V3 sequences exclusively direct viral CCR5 usage (Fig. 2C). The distinct V3 configuration in gp120 may contribute to the restriction of coreceptor type to be interacted, because amino acid residues at the V3 base directly participate in the binding to the N-terminal region of CCR5 [4]. In addition, structural differences among chemokine receptors may contribute. For example, the N-terminal region of CXCR4 is glycosylated, whereas that of CCR5 is not. The glycosylated V3 for neutralization resistance will sterically interrupt the access to the glycosylated coreceptor. Indeed, the removal of the N-linked glycosylation sites from CXCR4 allows the protein to serve as a universal coreceptor for both X4 and R5 viruses [39]. Further structural studies are under way to address this issue.

Our study has implications for HIV-1 vaccine design. The data suggests that a key impediment to the clinical use of Gp120 as an immunogen may be the cryptic nature of the R5 virus V3 neutralization epitope. A simple strategy to use R5 virus Gp120 will be insufficient even if the Gp120 of circulating HIV-1 subtype is carefully selected as immunogen. To develop strategies that could circumvent or overcome the impediment may be critical for practical application of Gp120 vaccine. In this regard, the amino acids that contribute to forming V3 conformation for the epitope masking through hydrogen bonds are relatively conserved among HIV-1 major subtypes in the world (Fig. 3D). Therefore, intervention in these interactions might be a target for a new strategy to improve effectiveness of immunological control of HIV-1.

In conclusion, we have identified here structural and functional features of HIV-1 CRF01_AE V3 elements those allow HIV-1 less sensitive to antibody neutralization. To our knowledge, this is the first report to show that the net positive charge of a neutralization epitope regulates viral sensitivity to humoral immunity. Thus, amino acid substitutions altering charged status of antigen site appear to deserve more attention, particularly in the adaptive evolution of HIV-1, as well as the other rapidly evolving pathogen.

## Materials and Methods

### Analysis of sequence diversity

Grouping of the sequences was done computationally using a software system, InforSense BioSense V3 (InforSense Ltd. http://www.inforsense.com). Nonsynonymous and synonymous nucleotide substitutions were calculated for all pair-wise sequence comparisons within each V3 subgroup using the Perl version of SNAP (Los Alamos HIV sequence database) according to the Nei and Gojobori method [40] incorporating the statistical method developed by Ota and Nei [41]. Amino acid variation at individual V3 positions was calculated according to the method described in the report by Huang et al [3] on the basis of Shannon's equation [42]:
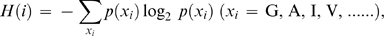
where *H(i)*, *p(x_i_)*, and *i* indicate the amino acid entropy score of a given position, the probability of occurrence of a given amino acid at the position, and the number of the position, respectively. An *H(i)* score of zero indicates absolute conservation, whereas 4.4 indicates complete randomness. The *H(i)* scores were expressed in the V3 sequence or in the three-dimensional structures constructed by the MD simulation method described below. The *p(x_i_)* scores were used to construct a consensus for each V3 structural group. Tajima's D statistic [32] for each type of V3 sequence population was calculated using DnaSP 4.10 [43].

### Blood specimens

Plasma samples were obtained from HIV-1 CRF01_AE positive individuals with written informed consent at Yokohama City University Hospital in Japan according to the rule of the ethics committee of the hospital. The clinical stages of the patients at the time of blood collection were A1 (*n* = 4), A2 (*n* = 4), B3 (*n* = 3), C2 (*n* = 1), C3 (*n* = 7), and unknown (*n* = 1) according to the 1993 Revised Classification System (CDC, USA). The CD4^+^ T-cell counts and HIV-1 RNA levels ranged from 2×10^3^ to 3719×10^3^ /ml blood (mean = 243×10^3^ /ml) and from <50 to 7.5×10^5^ copies/ml blood (mean = 2.4×10^4^ copies/ml), respectively. All plasma samples were heat-inactivated at 56°C for 30 minutes prior to use.

### Anti-V3 antibody titration

V3-peptide-based ELISA [35] was carried out using synthetic peptides matching to the central 19 amino acids of the V3 regions of the recombinant viruses (Fig. S3). Synthetic peptides were coated on 96-well plates (Immulon II; Dynatech Laboratories, Virginia, USA) and reacted with diluted plasma samples (1/10^4^). Antibodies bound to the peptides were detected with anti-human IgG peroxidase conjugate and 3,3′,5,5′-tetramethylbenzidine substrate (TMB peroxidase EIA substrate Kit, Bio-Rad Laboratories, USA). Each plasma sample was tested in duplicate.

### Neutralization assays

Plasmid DNAs containing HIV-1 V3 recombinant proviruses (*n* = 30) were constructed by the overlap extension method [13], [30], [33]. Cell-free viruses were prepared by transfection of the plasmid DNAs into HeLa cells as described previously [13], [30], [33]. Neutralization activities were measured in a single-round viral infectivity assay using CD4^+^CXCR4^+^CCR5^+^ HeLa cells [34]. Equal infectious titers of viruses (300 blue-cell-forming units) were incubated with serially diluted plasma samples (1/10 to 1/10^3^) for 60 min at 37°C. The infected cells were cultured for 48 hours at 37°C, fixed, and stained with 5-bromo-4-chloro-3-indolyl-β-D-galactopyranoside. Each plasma dilution was tested in duplicate, and the means of the positive blue cell numbers were used to calculate the 50% inhibition dose of viral infectivity (ND_50_). For plasma samples that did not neutralize a virus at the lowest dilution tested (1:10), an arbitrary titer of 1:5 (50 ND_50_ /ml) was used. In some cases, neutralization activities were measured using a multiple-round viral infectivity assay using NP-2 cell lines [44]. Equal infectious titers of the viruses (100 tissue culture infectious dose) were incubated with serially diluted plasma samples (1/10 to 1/10^3^) for 60 min at 37°C and used to infect the CD4^+^CXCR4^+^ NP-2 cells and CD4^+^CCR5^+^ NP-2 cells. After 60 min, the cells were washed once with phosphate-buffered saline. Culture supernatants were collected at 5 days after infection, and amounts of HIV-1 Gag p24 proteins were measured with a commercially available kit (RETROtek HIV-1 p24 Antigen ELISA, ZeptoMetrix Corporation, USA). Each plasma dilution was tested in duplicate, and the means of the p24 amounts were used to calculate the ND_50_.

### HIV-1 coreceptor usages

Previous data of the coreceptor tropisms of the recombinant viruses [13], [30], [33] were used.

### MD simulation

Gp120 outer domain structures bearing various V3 elements were constructed with the homology modelling technique, using the Molecular Operating Environment, MOE 2006.08 (Chemical Computing Group Inc., Montreal, Quebec, Canada) as described [45], [46]. As the modelling template, we used the crystal structure of HIV-1 gp120 containing an entire V3 element at a resolution of 3.30Å (PDB code: 2B4C), which represents the structure after the CD4 binding [3]. The 251amino-terminal and 24 carboxyl-terminal residues were deleted to construct the gp120 outer domain structure. MD simulations were performed using the SANDER module in the AMBER 8 program package [47] with MDGRAPE-3 (http://mdgrape.gsc.riken.jp/) and the AMBER parm99 force field with the TIP3P water model. After heating calculations for 20 picoseconds until 310 K using the NVT ensemble, the simulations were executed using the NPT ensemble at 1 atm and at 310 K for 5 nanoseconds. Superimpositions of the structures were done by coordinating atoms of amino acids along the β-sheet at the V3 base.

## Supporting Information

Figure S1 Information on the V3 sequences for the diversity analyses. Shown are the % distributions of CRF01_AE V3 sequences used in the present study (*n* = 1361) as a function of sampling years (a), countries (b), and V3 structural group (c). The sequences during 1991 to 2005 (*n* = 1148) represent a majority. They are mostly from Asia (15 countries, 1219 sequences). Others are from Africa (7 countries, 52 sequences), Europe (10 countries, 47 sequences), other regions (5 countries, 36 sequences), and unknown (1 sequence). V3 groups having CCR5 tropism (2b, 3b and 4b) represent the majority independent of the sampling period.
(0.34 MB TIF)

Figure S2 V3 diversity of HIV-1 subtypes A, B, and C. Global distribution (left) and _dn_/_ds_ ratios (right) of V3 structural variants of HIV-1 subtypes A, B, and C were examined, using the HIV-1 public database information from June 2007, and plotted as described in Fig. 1C.
(0.31 MB TIF)

Figure S3 V3 amino acid sequences of the recombinant viruses. V3 sequences of the recombinant viruses are from CRF01_AE clones in uncultured peripheral blood mononuclear cells from a Japanese family [30] (V3 IDs of A1{similar, tilde operator }A9 and B1{similar, tilde operator }B13), A1 variants having naturally occurring basic amino acid substitutions (mt1{similar, tilde operator }mt8) [13], and TH09 isolate having the CRF01_AE consensus V3 sequence [33] (TH09). Deduced amino acids of the V3 sequences were aligned with the CRF01_AE consensus sequence, ENSI-c. The small blue open box indicates a potential N-linked glycosylation site conserved in the V3 structural group b. Red letters indicates basic amino acid substitutions with respect to ENSI-c. The large black box indicates 19 amino acid sequences used for V3-peptide ELISA in Fig. 2. The net charge is the number of positively charged amino acids (R, K, and H) minus the number of negatively charged amino acids (D and E). Coreceptor tropism of the recombinant viruses was determined using CD4^+^CXCR4^+^ HOS cells and CD4^+^CCR5^+^ HOS cells [13], [30], [33].
(0.39 MB TIF)

Figure S4 Effects of protein G on plasma neutralizing activities. The plasma samples (YM17 and YM61) were incubated with serially diluted protein G agarose solution (GammaBind Plus Sepharose, Amersham) for 60 min at 37°C. The agarose was removed by brief centrifugation, and the supernatants were used to measure ND_50_ against LAI recombinant viruses having non-rV3 (B1 and B10) using CD4^+^CXCR4^+^CCR5^+^ HeLa cells (MAGIC-5 cells [34]) as described in Materials and Methods.
(0.21 MB TIF)

Figure S5 Antibody epitope mapping of the rV3. Peptide-based, enzyme-linked immunosorbent assay [35] was carried out using indicated synthetic peptides matching the rV3 amino acids of the recombinant viruses (SI Fig. 3, recombinant ID of A1). Antibodies bound to the peptides were detected with anti-human IgG peroxidase conjugate and 3,3′,5,5′-tetramethylbenzidine substrate. Absorbance at 450 nm is shown.
(0.27 MB TIF)

Figure S6 ND_50_ in the single- and multiple-round viral infectivity assays. Plasma samples (*n* = 8) were used to measure ND_50_ against LAI recombinant viruses having rV3 (clone IDs of ENSI-c and A1) and non-rV3 (B6). The ND_50_ were measured in a single-round viral infectivity assay using CD4^+^CXCR4^+^CCR5^+^ HeLa cells (MAGIC-5 cells) [34] or a multiple-round viral infectivity assay using CD4^+^CXCR4^+^ NP2 cells and CD4^+^CCR5^+^ NP2 cells (NP-2 cells) [44] as described in Materials and Methods. Red diamonds indicate the medians of the neutralization titers of the 8 plasma samples.
(0.20 MB TIF)

Figure S7 Close-up view of the V3 base-stem region of Gp120 with non-rV3. LAI Gp120 outer domain structures with B1 V3 were constructed computationally by methods of homology modelling and molecular dynamic simulation at a simulation time of 5 nanoseconds.
(0.64 MB TIF)

Table S1 (0.04 MB PDF)

Table S2 (0.04 MB PDF)
