# Supplementary figures and images for: Net Positive Charge of HIV-1 CRF01_AE V3 Sequence Regulates Viral Sensitivity to Humoral Immunity
Source: PLoS One. 2008 Sep 12;3(9):e3206. doi: 10.1371/journal.pone.0003206 (PMC2527523; doi:10.1371/journal.pone.0003206)

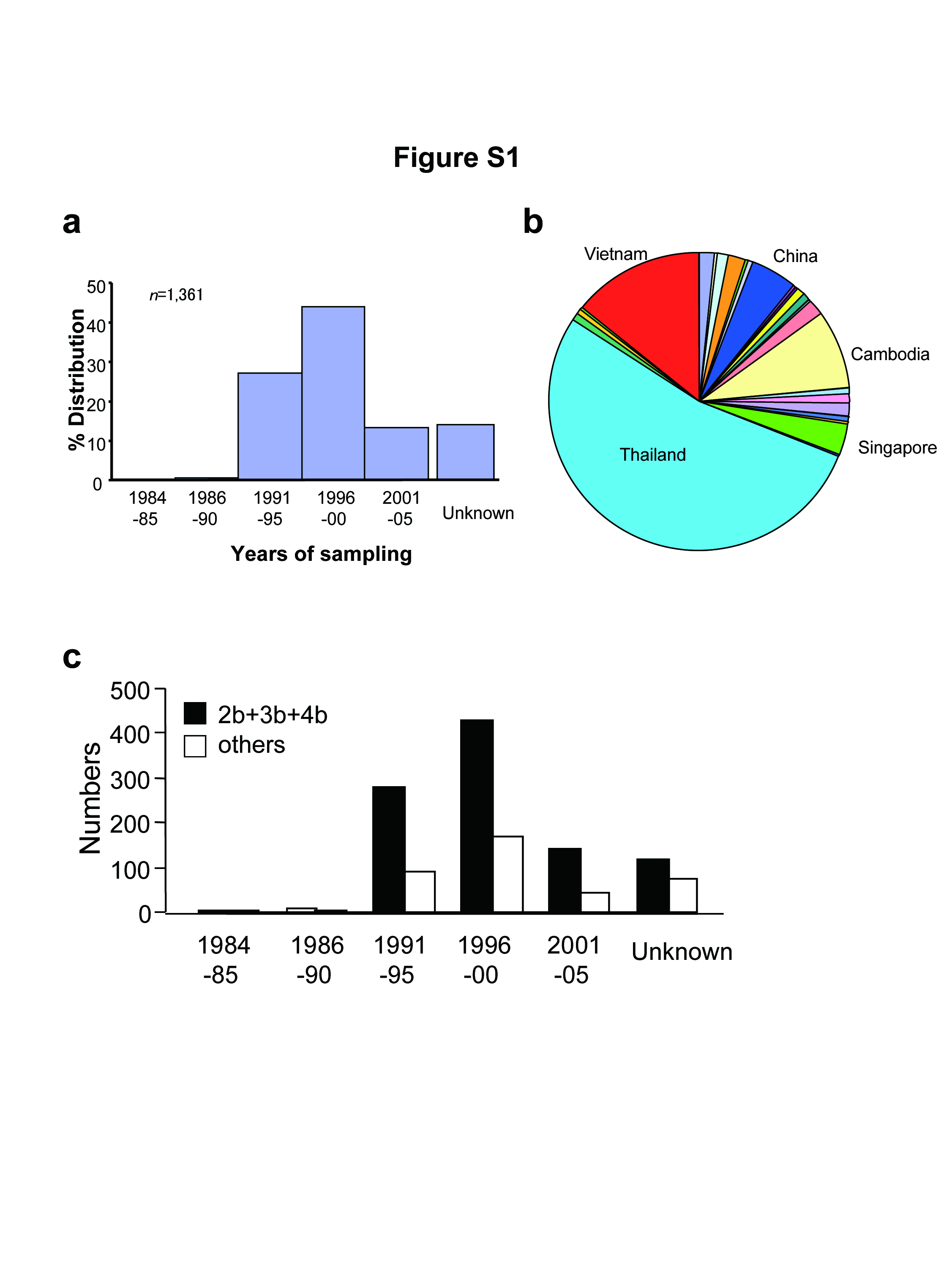

Supplement: Figure S1 — Information on the V3 sequences for the diversity analyses. Shown are the % distributions of CRF01_AE V3 sequences used in the present study (n = 1361) as a function of sampling years (a), countries (b), and V3 structural group (c). The sequences during 1991 to 2005 (n = 1148) represent a majority. They are mostly from Asia (15 countries, 1219 sequences). Others are from Africa (7 countries, 52 sequences), Europe (10 countries, 47 sequences), other regions (5 countries, 36 sequences), and unknown (1 sequence). V3 groups having CCR5 tropism (2b, 3b and 4b) represent the majority independent of the sampling period. (0.34 MB TIF) [file pone.0003206.s001.tif]

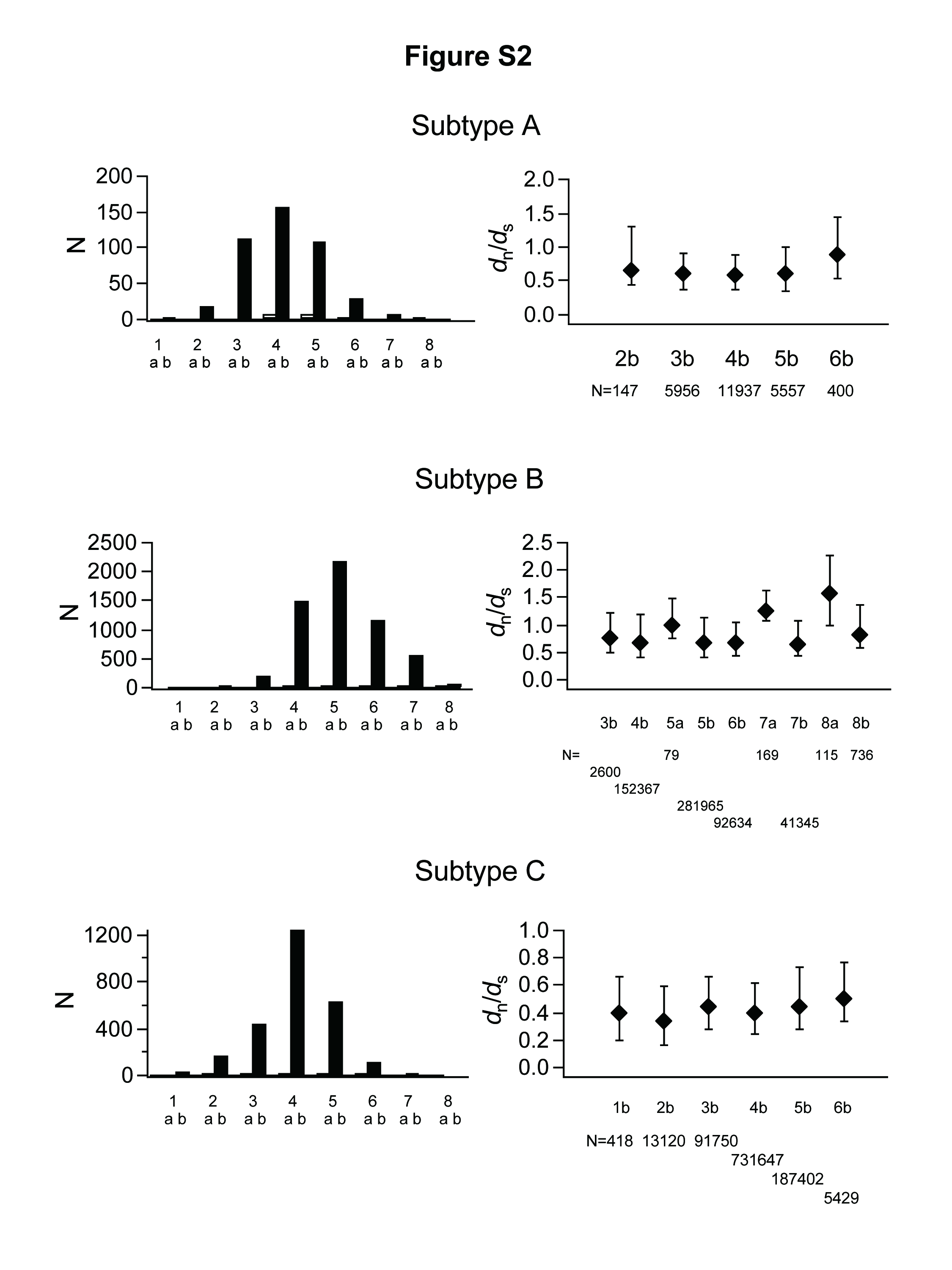

Supplement: Figure S2 — V3 diversity of HIV-1 subtypes A, B, and C. Global distribution (left) and dn/ds ratios (right) of V3 structural variants of HIV-1 subtypes A, B, and C were examined, using the HIV-1 public database information from June 2007, and plotted as described in Fig. 1C. (0.31 MB TIF) [file pone.0003206.s002.tif]

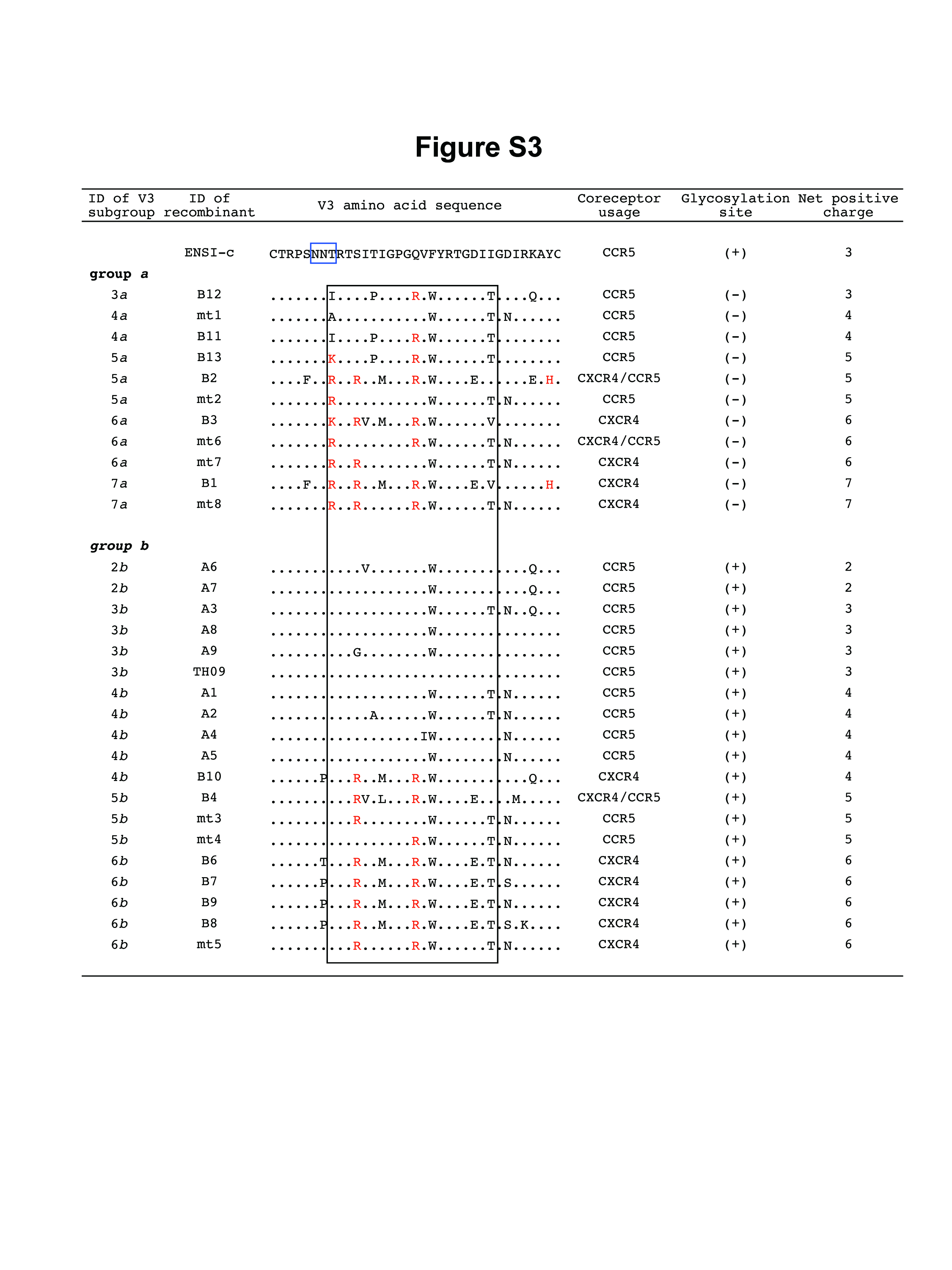

Supplement: Figure S3 — V3 amino acid sequences of the recombinant viruses. V3 sequences of the recombinant viruses are from CRF01_AE clones in uncultured peripheral blood mononuclear cells from a Japanese family [30] (V3 IDs of A1{similar, tilde operator }A9 and B1{similar, tilde operator }B13), A1 variants having naturally occurring basic amino acid substitutions (mt1{similar, tilde operator }mt8) [13], and TH09 isolate having the CRF01_AE consensus V3 sequence [33] (TH09). Deduced amino acids of the V3 sequences were aligned with the CRF01_AE consensus sequence, ENSI-c. The small blue open box indicates a potential N-linked glycosylation site conserved in the V3 structural group b. Red letters indicates basic amino acid substitutions with respect to ENSI-c. The large black box indicates 19 amino acid sequences used for V3-peptide ELISA in Fig. 2. The net charge is the number of positively charged amino acids (R, K, and H) minus the number of negatively charged amino acids (D and E). Coreceptor tropism of the recombinant viruses was determined using CD4+CXCR4+ HOS cells and CD4+CCR5+ HOS cells [13], [30], [33]. (0.39 MB TIF) [file pone.0003206.s003.tif]

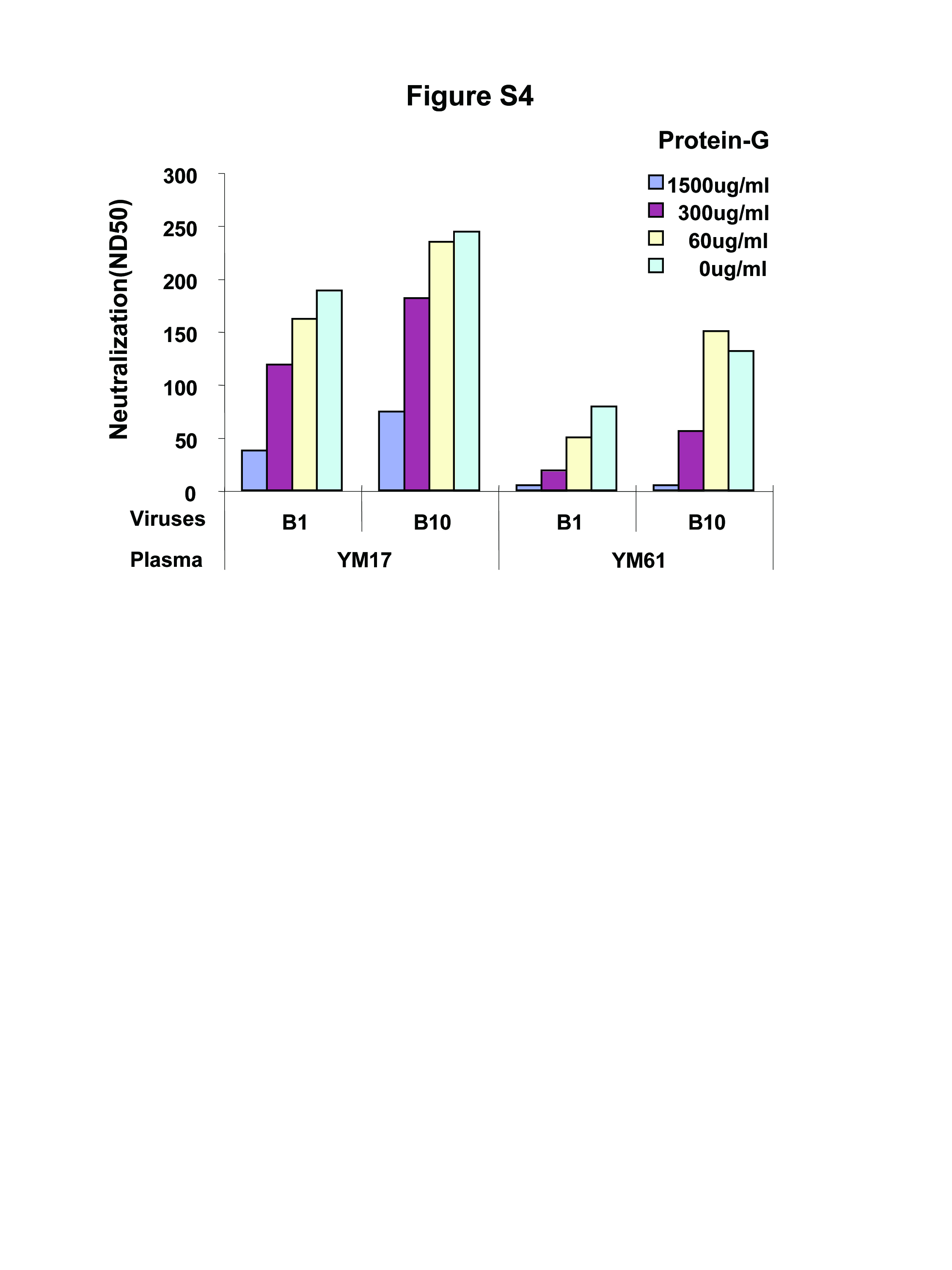

Supplement: Figure S4 — Effects of protein G on plasma neutralizing activities. The plasma samples (YM17 and YM61) were incubated with serially diluted protein G agarose solution (GammaBind Plus Sepharose, Amersham) for 60 min at 37°C. The agarose was removed by brief centrifugation, and the supernatants were used to measure ND50 against LAI recombinant viruses having non-rV3 (B1 and B10) using CD4+CXCR4+CCR5+ HeLa cells (MAGIC-5 cells [34]) as described in Materials and Methods. (0.21 MB TIF) [file pone.0003206.s004.tif]

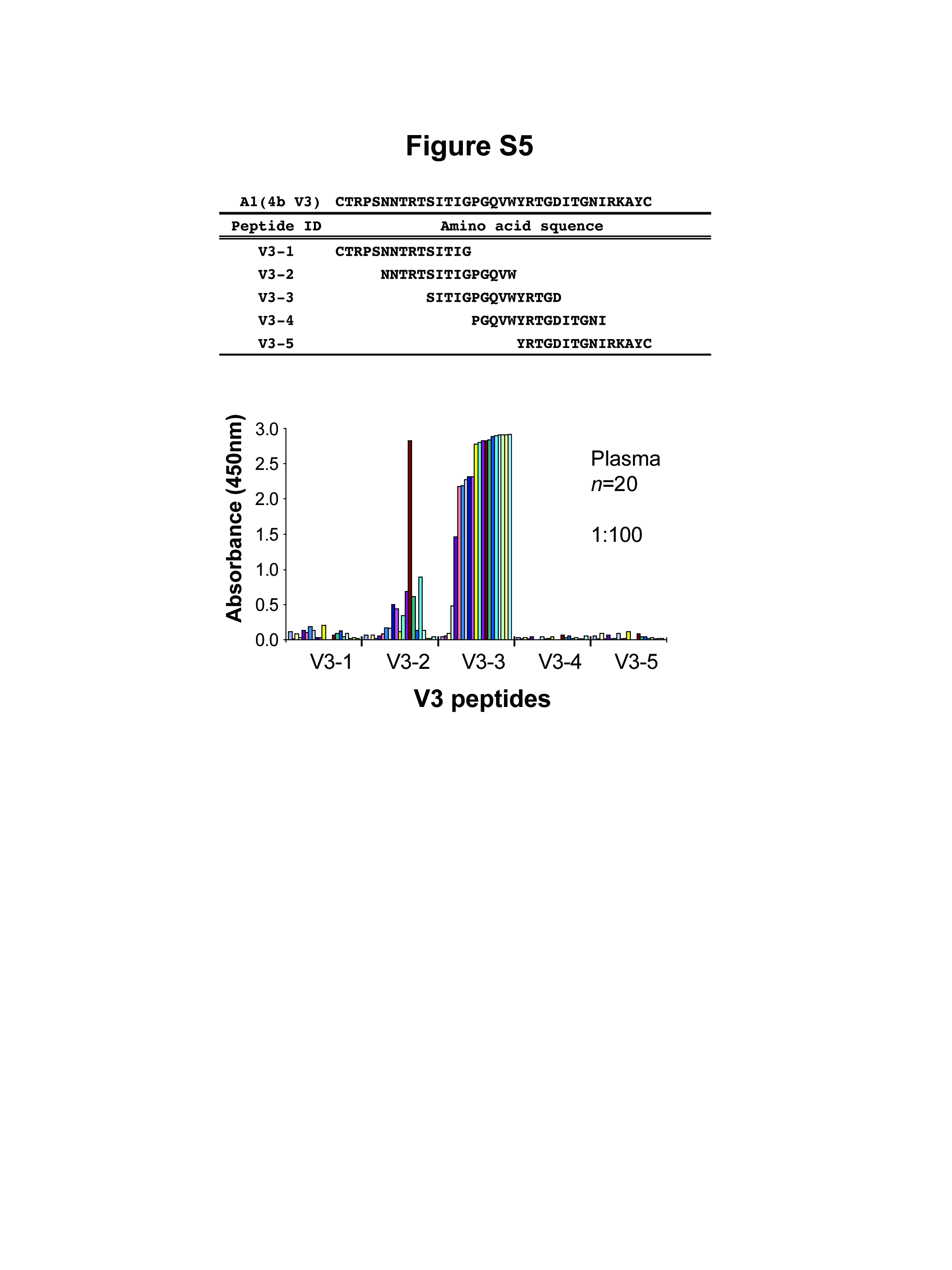

Supplement: Figure S5 — Antibody epitope mapping of the rV3. Peptide-based, enzyme-linked immunosorbent assay [35] was carried out using indicated synthetic peptides matching the rV3 amino acids of the recombinant viruses (SI Fig. 3, recombinant ID of A1). Antibodies bound to the peptides were detected with anti-human IgG peroxidase conjugate and 3,3′,5,5′-tetramethylbenzidine substrate. Absorbance at 450 nm is shown. (0.27 MB TIF) [file pone.0003206.s005.tif]

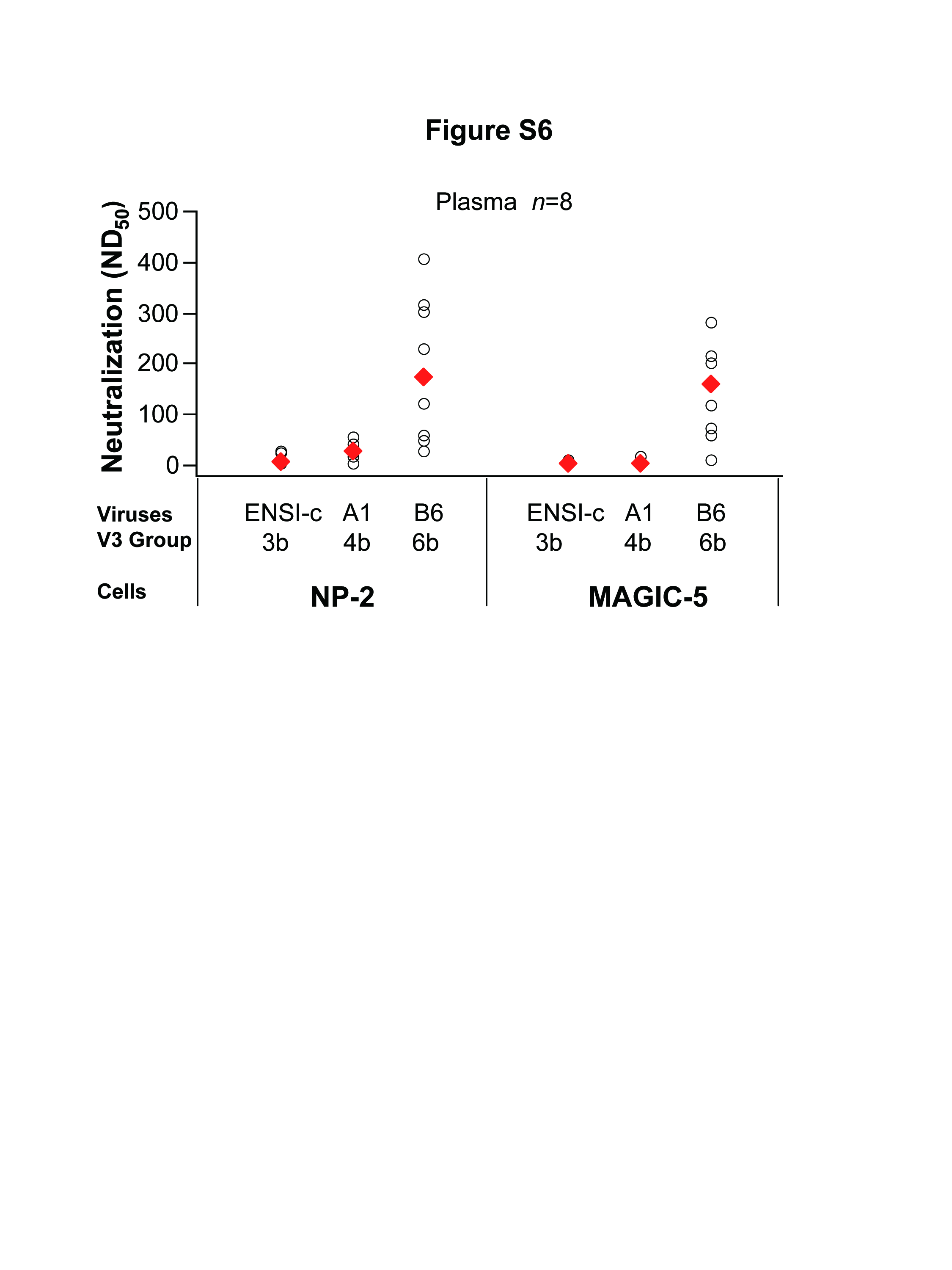

Supplement: Figure S6 — ND50 in the single- and multiple-round viral infectivity assays. Plasma samples (n = 8) were used to measure ND50 against LAI recombinant viruses having rV3 (clone IDs of ENSI-c and A1) and non-rV3 (B6). The ND50 were measured in a single-round viral infectivity assay using CD4+CXCR4+CCR5+ HeLa cells (MAGIC-5 cells) [34] or a multiple-round viral infectivity assay using CD4+CXCR4+ NP2 cells and CD4+CCR5+ NP2 cells (NP-2 cells) [44] as described in Materials and Methods. Red diamonds indicate the medians of the neutralization titers of the 8 plasma samples. (0.20 MB TIF) [file pone.0003206.s006.tif]

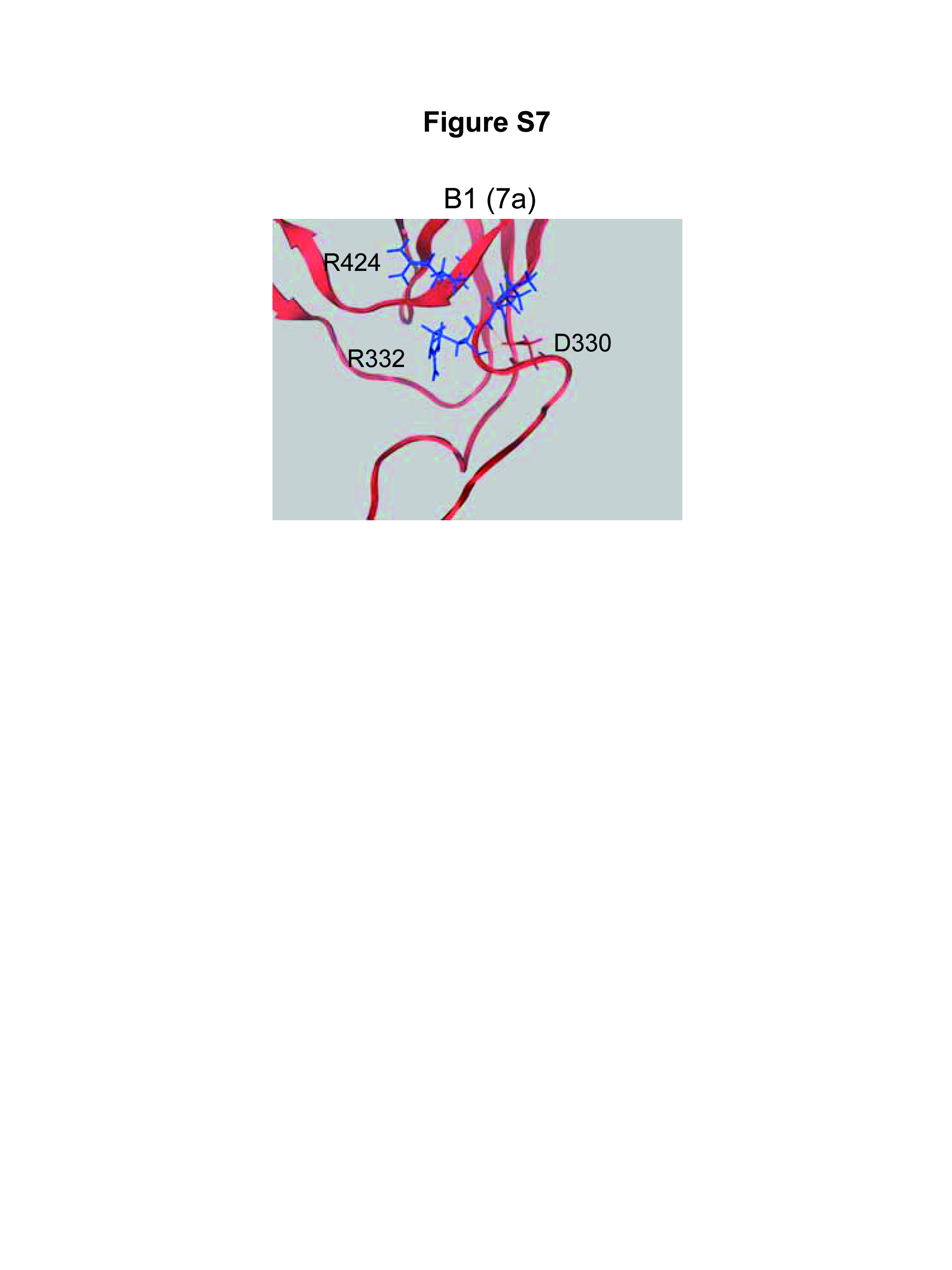

Supplement: Figure S7 — Close-up view of the V3 base-stem region of Gp120 with non-rV3. LAI Gp120 outer domain structures with B1 V3 were constructed computationally by methods of homology modelling and molecular dynamic simulation at a simulation time of 5 nanoseconds. (0.64 MB TIF) [file pone.0003206.s007.tif]
