# Supplementary material for: Net Positive Charge of HIV-1 CRF01_AE V3 Sequence Regulates Viral Sensitivity to Humoral Immunity
Source: PLoS One. 2008 Sep 12;3(9):e3206. doi: 10.1371/journal.pone.0003206 (PMC2527523; doi:10.1371/journal.pone.0003206)
Supplement: Table S1 — (0.04 MB PDF) [file pone.0003206.s008.pdf]

**Table S1. Mann-Whitney U test of $d_n/d_s$ ratios for each type of V3 structure.**

| | vs. | *n1* | *n2* | U | *P* |
|---|---|---|---|---|---|
| 3a | 3b | 533 | 120,133 | 23,469,516 | <0.001 |
| 4a | 4b | 2,208 | 35,813 | 32,552,854 | <0.001 |
| 5a | 5b | 2,240 | 37,28 | 2,955,175 | <0.001 |
| 6a | 6b | 1,685 | 27 | 17,206 | 0.0015 |
| | | | | | |
| 3a | 4a | 533 | 2,208 | 414,835 | <0.001 |
| 4a | 5a | 2,208 | 2,240 | 2,111,520 | <0.001 |
| 5a | 6a | 2,240 | 1,685 | 1,755,988 | <0.001 |
| 6a | 7a | 1,685 | 208 | 149,850 | <0.001 |
| | | | | | |
| 2b | 3b | 4,433 | 120,133 | 189,757,552 | <0.001 |
| 3b | 4b | 60,179 | 17,825 | 337,417,856 | <0.001 |
| 4b | 5b | 35,813 | 3,728 | 64,184,132 | <0.001 |
| 5b | 6b | 3,728 | 27 | 49,880 | 0.468 |

*n1*, *n2*: Number of sequence pairs, U: Mann-Whitney's U statistics, *P*: Level of significance
