# Supplementary material for: Net Positive Charge of HIV-1 CRF01_AE V3 Sequence Regulates Viral Sensitivity to Humoral Immunity
Source: PLoS One. 2008 Sep 12;3(9):e3206. doi: 10.1371/journal.pone.0003206 (PMC2527523; doi:10.1371/journal.pone.0003206)
Supplement: Table S2 — (0.04 MB PDF) [file pone.0003206.s009.pdf]

Table S2. Tajima's D statistic values for each type of V3 structure

class	m	S	Eta	π	θ_κ	D	significance
2b	103	67	116	0.0689	0.2122	-2.2264	p<0.01
3a	34	65	96	0.117	0.2236	-1.785	ns
3b	577	81	155	0.0498	0.2128	-2.2441	p<0.01
4a	69	87	154	0.1401	0.3082	-1.8889	p<0.05
4b	298	86	154	0.0746	0.2338	-2.0792	p<0.05
5a	69	86	153	0.1518	0.3062	-1.7455	ns
5b	88	79	125	0.1137	0.2358	-1.7386	ns
6a	59	72	125	0.1546	0.2562	-1.3913	ns
6b-9a	51	79	144	0.1823	0.3048	-1.439	ns

m: Number of sequences, S: Number of segregating sites, Eta: Total number of mutations

π: nucleotide diversity, θ_κ: q (=4Neu) per site calculated from Eta, D: Tajima's D statistic value [32]
